# Supplementary material for: Composition of Carotid Plaques Differs Between Chinese and United States Patients: A Histology Study
Source: Res Sq. 2025 Jul 14:rs.3.rs-7005564. Preprint. [Version 1] doi: 10.21203/rs.3.rs-7005564/v1 (PMC12288538; doi:10.21203/rs.3.rs-7005564/v1)
Supplement: Supplement 1 [file NIHPPrs7005564v1-supplement-1.pdf]

Supplement Figure 1

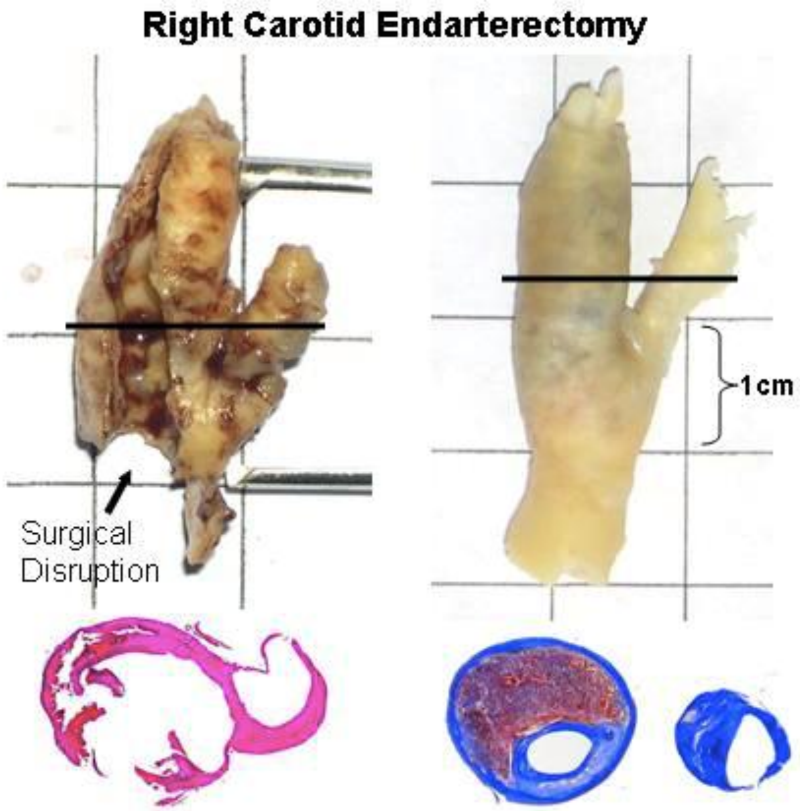

Left. Carotid plaque from a standard endarterectomy with disruption of the specimen. Right. Specimen from the modified surgical technique without disruption of the inner surface. Corresponding histologic sections are below.

Supplement Figure 2

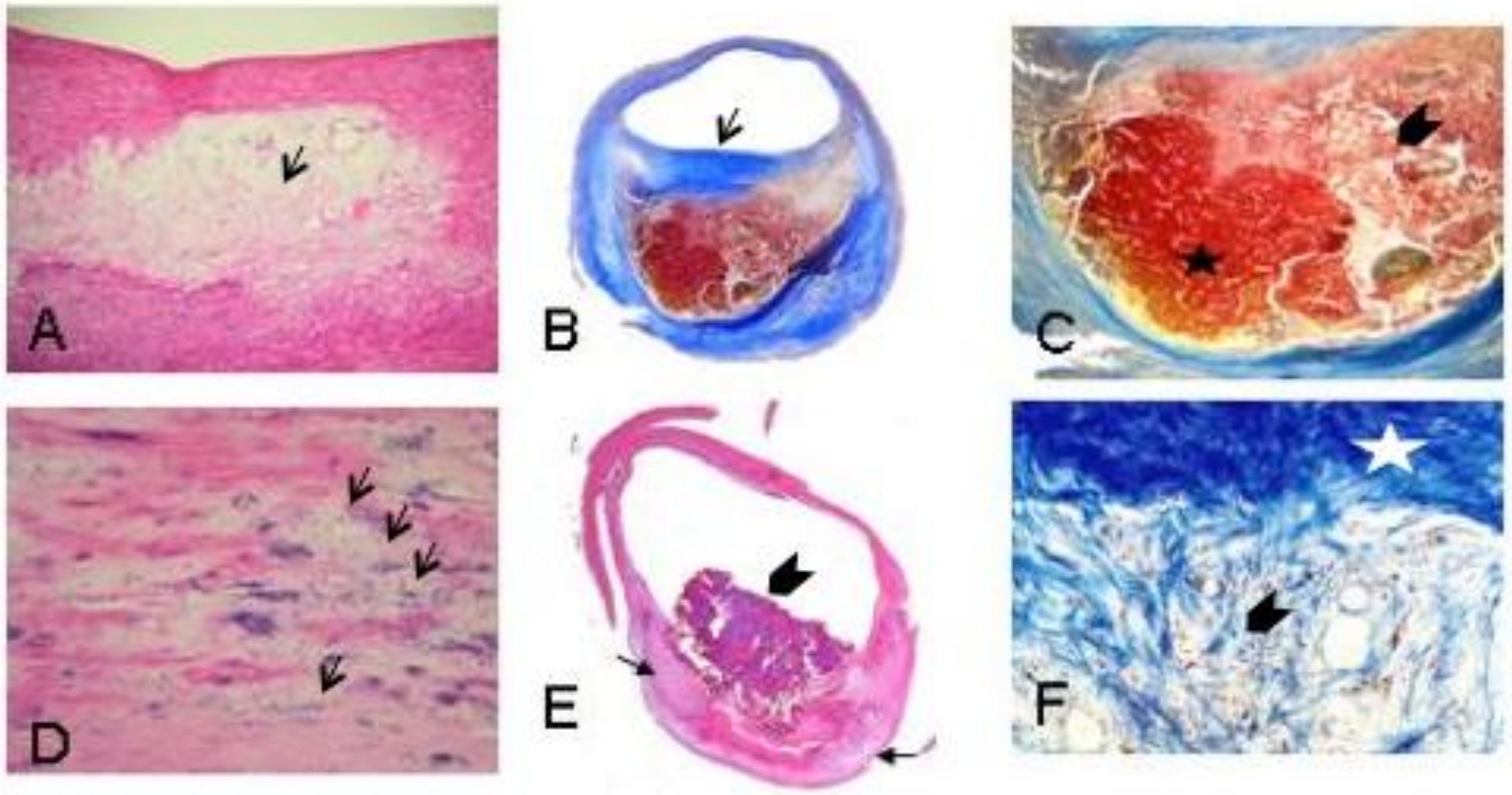

Carotid plaque components: A) Lipid pool (arrow)(H&E); B) Necrotic core with thick fibrous cap (arrow) (Mallory's trichrome); C) Recent intraplaque hemorrhage into a necrotic core (star) (Mallory's trichrome). Late intraplaque hemorrhage in the necrotic core (chevron) (Mallory's trichrome); D) Speckled calcification (arrows)(H&E); E) Protruding calcium nodule (chevron) with two calcified plates (arrows)(H&E); F) Loose matrix (chevron) with adjacent dark blue dense matrix (star) (Mallory's trichrome).

Supplement Table 1

Correlation of plaque composition with stenosis and percent wall volume (N=186)

	Correlation with Stenosis			Correlation with Percent Wall Volume	
Component	Spearman's r	p		Spearman's r	p
Lipid pool	0.30	<0.001		0.23	0.002
Necrotic core	0.30	<0.001		0.34	<0.001
Hemorrhage, %	0.29	<0.001		0.39	<0.001
Recent hemorrhage, %	0.30	<0.001		0.35	<0.001
Late hemorrhage, %	0.00	0.98		0.15	0.048
Calcification, %	-0.25	0.001		0.06	0.44
Loose matrix, %	0.25	0.001		0.15	0.044
